# Platelet factor 4 in cognitive, immune and hematopoietic aging: emerging evidence and translational challenges

**DOI:** 10.3389/fimmu.2026.1883921

**Published:** 2026-09-02

**Authors:** Yingying Guo, Zihua Liu, Weiwei Lu, Hao Zhuo, Zhicheng Wang, Xiaoning Wang

**Affiliations:** 1 Department of Blood Transfusion, The First Hospital of Jilin University, Changchun, Jilin, China; 2 Department of Blood Transfusion Service, The Second Hospital of Lanzhou University, Lanzhou, Gansu, China; 3 Department of Transfusion Medicine, Huashan Hospital, Fudan University, Shanghai, China

**Keywords:** aging-associated pathology, cognitive impairment, CXCR3, hematopoietic stem cells, immunosenescence, neuroinflammation, platelet factor 4

## Abstract

Platelet Factor 4 (PF4) is a CXC chemokine abundantly sequestered within platelet α-granules. Its modulatory roles in age-related dysfunction across cognitive, immune, and hematopoietic systems have garnered increasing research attention in recent years. PF4 modulates an array of biological pathways associated with the attenuation of aging-related cellular defects; these pathways are activated by physical activity, young plasma exposure, and Klotho-related signaling cascades. Mechanistically, PF4 engages CXCR3 and LDLR to activate downstream PI3K/Akt and MAPK signaling axes, exerting neuroprotective and immunomodulatory effects and restoring function in aged hematopoietic stem cells (HSCs). Collectively, these actions exert multifaceted effects on aging-associated cellular dysfunction. Within the central nervous system, PF4 mitigates hippocampal neuroinflammation, enhances synaptic plasticity, and promotes adult hippocampal neurogenesis to ameliorate age-dependent learning and memory deficits. In the peripheral immune system, PF4 restores balanced T-cell subset distribution and reduces circulating levels of pro-senescent inflammatory mediators. Within the bone marrow hematopoietic niche, PF4 preserves HSC quiescence, facilitates DNA damage repair, and maintains lymphoid differentiation potential. Clinical observational studies have identified correlations between circulating PF4 levels and multiple age-associated pathologies, including Alzheimer’s disease, sarcopenia, stroke, and coronary artery disease, suggesting its potential as an adjunct diagnostic biomarker. Nevertheless, published findings regarding age-related changes in PF4 levels remain inconsistent, largely due to heterogeneous sample preparation protocols and detection methodologies. Furthermore, PF4 carries inherent thrombotic, profibrotic, and autoantigenic risks. Unresolved questions surrounding long-term safety, standardized dosing regimens, and tissue-targeted delivery systems represent major barriers to clinical translation. This review systematically delineates the multilayered modulatory mechanisms of PF4 in age-related cognitive, immune, and hematopoietic dysfunction, as well as its translational prospects, unresolved discrepancies, and key research gaps. We further outline prospective research directions, including protein structural optimization, refined targeted delivery strategies, and combinatorial intervention regimens, to establish a theoretical framework for future preclinical studies of PF4 as a modulator of discrete aging-related cellular phenotypes.

## 1 Introduction

Global population aging is progressing at an unprecedented rate. As the proportion of older adults continues to rise, the public health burden attributable to aging-associated diseases is steadily escalating (1).Aging represents the strongest known risk factor for neurodegenerative diseases, including Alzheimer’s disease (AD) and Parkinson’s disease (PD), whose prevalence rises exponentially with advancing age. According to the 2024 Alzheimer’s Disease Facts and Figures report from the Alzheimer’s Association, AD prevalence increases from approximately 5% in adults aged 65–74 years to more than 33% in those aged 85 years and older, underscoring the substantial public health impact of aging-related neurodegeneration (2).

Aging arises from cumulative multi-layered biological alterations. A landmark 2025 updated framework of aging hallmarks expanded the original 12 canonical axes to 14 core categories, namely genomic instability, telomere attrition, epigenetic remodeling, impaired proteostasis, defective macroautophagy, dysregulated nutrient sensing, mitochondrial dysfunction, cellular senescence, stem cell exhaustion, aberrant intercellular crosstalk, chronic low-grade inflammation, microbial dysbiosis, extracellular matrix remodeling, and psychosocial disconnection (3).These hallmarks are interconnected and mutually reinforcing, collectively driving progressive functional decline at the organismal level. Accordingly, interventions targeting the aging process itself rather than focusing exclusively on individual pathological proteins are emerging as promising therapeutic strategies (4).

Over the past two decades, heterochronic parabiosis studies have provided foundational evidence for the tissue-protective effects of youthful systemic circulation. Pioneering work demonstrated that exposure to a young circulatory niche restores regenerative capacity in muscle stem cells and hepatic progenitor cells of aged mice (5). Subsequent investigations extended these effects to multiple organ systems: youthful circulating factors promote remyelination in the aged central nervous system (6), drive cerebrovascular remodeling, boost hippocampal neurogenesis, and restore olfactory discrimination in aged rodents (7). Intravenous administration of young plasma also markedly improves contextual fear memory and spatial learning performance in aged mice (8). These findings have stimulated research into circulating youth-associated mediators and prompted early-phase clinical trials for AD and PD (9, 10). However, direct young blood infusion raises ethical concerns, limited donor supply, risks of immune rejection, and pathogen transmission. Accordingly, the field has focused on identifying specific circulating molecules that mitigate aging-related functional deficits.

Platelet factor 4 (PF4) has emerged precisely in this context. Three independent research groups published complementary preclinical findings in 2023 identifying PF4’s modulatory roles in aging-associated pathologies. Circulating PF4 concentrations are markedly higher in young versus aged plasma, and systemic delivery of recombinant PF4 mitigates hippocampal neuroinflammation and improves cognitive function in aged mice, suggesting PF4 functions as one effector of young plasma signaling (11). Another study revealed that treatment with the longevity protein Klotho activates platelets and increases plasma PF4 levels; peripherally administered PF4 can be detected in brain tissue and directly enhance hippocampal synaptic plasticity, supporting PF4 as a partial intermediate linking physical activity to improved neuronal maturation (12). A third body of preclinical work demonstrates that acute physical activity triggers platelet activation and subsequent PF4 secretion; the ablation of PF4 eliminates exercise-associated hippocampal neurogenesis in knockout mice, implying PF4 may act as one contributory factor linking physical activity to enhanced neurogenesis (13). Together, studies focusing on Klotho, exercise, and young blood indicate PF4 abundance is modulated by interventions that mitigate aging-related deficits. Circulating PF4 levels tend to decline with advancing age, and exogenous PF4 delivery can partially replicate several favorable phenotypes linked to the three interventions in preclinical experiments. Such results support PF4 as a candidate target to explore compounds that reproduce limited exercise-derived protective effects (14).

This review systematically synthesizes recent progress in PF4 geroscience research. By integrating key preclinical and clinical observations from the past several years, we conduct a multidimensional analysis encompassing PF4’s molecular signaling cascades, systemic tissue effects, translational prospects, and unresolved safety limitations. We further outline prospective research avenues for PF4 as an agent that partially recapitulates exercise-associated neuroprotective benefits, rather than a broad-spectrum anti-aging therapeutic.

## 2 Molecular basis and functions of PF4

### 2.1 Cellular sources and expression regulation of PF4

PF4 is predominantly produced by bone marrow megakaryocytes and sequestered within platelet α-granules, representing one of the most abundant proteins within these organelles. Approximately 20 μg PF4 is stored per 10^9^ human platelets, while steady-state plasma PF4 concentrations only span 2–10 ng/mL (15). Following platelet activation, PF4 is rapidly released from α-granules via a P-selectin-dependent mechanism, generating localized high-concentration signaling niches. Beyond megakaryocyte-derived platelets, recent work has identified PF4 production in macrophages and activated T lymphocytes (16).

PF4 transcription is tightly controlled by a panel of transcription factors. A conserved -51 ETS (E26 transformation-specific) binding motif within the PF4 promoter serves as a docking site for FLI-1, ELF-1, and GABP, which coordinately drive PF4 gene expression. FLI-1 synergizes with GATA-1, a master regulator of megakaryocyte lineage specification, to transactivate the PF4 promoter and shows upregulated expression across all stages of megakaryocyte maturation (17).Runt-related transcription factor 1 (RUNX1) acts as a transcriptional modulator of PF4; RUNX1 haploinsufficiency results in diminished PF4 transcript and protein abundance (18). ETS transcription factor 4 also transactivates PF4 transcription to support host innate defense against Plasmodium pathogens (19).

### 2.2 Receptor spectrum and signal transduction mechanisms of PF4

PF4 elicits diverse biological outputs via engagement with multiple cell-surface receptors, and this broad receptor repertoire underpins its pleiotropic bioactivity (16). C-X-C motif chemokine receptor 3 (CXCR3) represents the best-characterized PF4 binding partner. Alternative splicing generates two primary CXCR3 isoforms: CXCR3A and CXCR3B. PF4 exhibits high binding affinity for CXCR3B, and this interaction suppresses endothelial proliferation and angiogenic signaling. Within immune populations, PF4-CXCR3A ligation drives Gαi-dependent chemotaxis of activated T cells, with downstream signaling cascades encompassing MAPK and PI3K/Akt axes (20, 21). PF4-CXCR3 signaling further activates the PI3K/AKT/Nrf2 cascade to mitigate neuronal ferroptosis in murine intracerebral hemorrhage models and PC12 cell cultures; pharmacological blockade of CXCR3, PI3K, or Nrf2 abolishes this neuroprotective phenotype. CXCR3 is abundantly expressed across peripheral immune subsets yet minimally detected in brain parenchymal cells, establishing a molecular framework through which PF4 modulates central nervous system function indirectly via peripheral immune remodeling.

Low-density lipoprotein receptor (LDLR) constitutes another vital PF4 receptor. PF4-LDLR binding disrupts LDL endocytosis, leading to cell-surface LDL retention—a mechanism implicated in atherosclerotic progression (23). Work from Zhang et al. further validated LDLR as a primary receptor transducing PF4’s modulatory signals in HSCs; PF4 blunts LDL uptake through LDLR to reprogram HSC metabolic status and proliferative activity (24).

Low-density lipoprotein receptor-related protein 1 (LRP1) also serves as a functional PF4 receptor. PF4 restrains megakaryocyte differentiation via LRP1 to establish a negative feedback circuit; genetic deletion of LRP1 restricted to megakaryocytes abrogates PF4-mediated suppression of megakaryopoiesis (25). The leukocyte integrin Mac-1 (CD11b/CD18) acts as an additional functional PF4 receptor: PF4 triggers adhesion and chemotaxis in Mac-1-expressing neutrophils and monocytes via direct binding to the αM I-domain of the integrin, and further augments phagocytic capacity in macrophages (26).

PF4 functions as an agonist for C-C motif chemokine receptor 1 (CCR1) to promote human monocyte chemotaxis (27). Additionally, PF4 neutralizes the anticoagulant capacity of activated protein C (APC), redirecting APC toward cytoprotective signaling at sites of platelet activation (28).

Collectively, PF4 interacts with a panel of receptors—CXCR3, LDLR, LRP1, Mac-1, and CCR1—to govern core biological processes including cell migration, lipid turnover, megakaryocyte maturation, phagocytosis, and anticoagulant signaling. This multifaceted receptor engagement lays the molecular foundation for PF4’s pleiotropic roles in hemostasis, immune homeostasis, angiogenesis, and hematopoiesis.

### 2.3 Homeostatic functions and aging-modulatory effects of PF4

PF4’s extensive receptor repertoire and distinct structural properties enable broad biological activity under physiological homeostatic conditions. Within hemostatic pathways, PF4 forms immunogenic complexes with heparin, which are the primary antigenic basis of heparin-induced thrombocytopenia (HIT). PF4 also binds platelet-surface c-Mpl to trigger JAK2-STAT3/STAT5 signaling and platelet aggregation, a cascade linked to vaccine-induced immune thrombotic thrombocytopenia (VITT) (29). As an immunomodulatory chemokine, PF4 governs neutrophil trafficking, monocyte differentiation, and the equilibrium between effector and regulatory T cell populations (30–32). PF4 displays context-dependent opposing functions during pathogenic infection: it supports host clearance of bacterial sepsis and pulmonary pathogens yet exacerbates neuroinflammation in cerebral malaria, highlighting its dual immunomodulatory capacity (33–36). PF4 additionally inhibits angiogenic signaling and functions as a homeostatic regulator to maintain HSC quiescence in the bone marrow microenvironment (37–39).

This body of literature constitutes the canonical understanding of PF4, centered on hemostasis, host immunity, and antimicrobial defense. While these canonical physiological roles have been well characterized for decades, accumulating preclinical evidence over recent years has uncovered a previously underappreciated layer of PF4 bioactivity related to aging modulation. However, accumulating preclinical datasets have greatly broadened our appreciation of PF4’s bioactivity by uncovering its tissue-protective impacts across a range of aging-linked pathological features. PF4 exerts tiered modulatory effects on discrete aged cell and tissue phenotypes spanning multiple organ systems: it attenuates age-dependent cognitive impairment, restores balanced peripheral immune architecture, and rectifies aging-associated HSC dysfunction. These paradigm-shifting findings have extended PF4 research beyond classical hemostasis and infectious immunology into the field of geroscience. Subsequent sections elaborate the mechanistic underpinnings of these aging-modulatory actions.

## 3 PF4 and age-related cognitive dysfunction

### 3.1 PF4 ameliorates age-associated cognitive deficits

Within cognitive geroscience, accumulating preclinical murine data indicate that PF4 ameliorates age-associated cognitive deficits. Schroer et al. performed a comprehensive series of behavioral assays documenting PF4’s cognitive-beneficial effects in aged male mice (11). Intravenous PF4 administration to 20-month-old rodents improved performance in novel object recognition, Y-maze, and radial arm water maze tasks; recombinant human PF4 exerted comparable effects, indicating evolutionary conservation of this phenotype across species. Middle-aged PF4-knockout mice show pronounced deficits in object recognition, spatial working memory, and associative learning, confirming that endogenous PF4 is necessary to maintain intact cognitive function and that PF4 deficiency accelerates age-related cognitive decline. This cognition-modulatory effect exhibits age selectivity in murine models: it induces broad improvements in aged mice but exerts minimal functional changes in young controls (13). Zhuo et al. provided a systematic overview of PF4’s cognition-modulating capacity, noting its ability to counteract aging-driven neuronal dysfunction (40). Xie et al. further reported that surgical anesthesia exacerbates hippocampal neuronal senescence in aged mice, while intraperitoneal PF4 delivery mitigates neuronal aging phenotypes, implying prospective utility of PF4 for perioperative neurocognitive preservation (41).

### 3.2 PF4 reduces hippocampal neuroinflammation

One of the hallmark features of the aged brain is chronic low-grade neuroinflammation, manifested by elevated expression of pro-inflammatory factors, complement system activation, and microglial activation (42). PF4 exerts its cognitive-improving effects by alleviating neuroinflammation, reversing age-related changes in the immune system, and restoring the expression of cognitive-related factors like synaptic plasticity-related proteins in the hippocampus to youthful levels (43).PF4 administration significantly downregulated hippocampal expression of pro-inflammatory factors including *Tnf, Nfkb1, Il1b, Clqb, and Cd11b* in aged mice (11). Immunohistochemical assays revealed a marked reduction in *C1q* protein levels in the aged mouse dentate gyrus, alongside an approximately 30% decrease in CD68^+^ activated microglia. Single-cell RNA sequencing further demonstrated that PF4 intervention shifted the transcriptional profile of aged hippocampal microglia toward a younger signature, with robust downregulation of TNF-mediated signaling, type I interferon signaling, and inflammatory mediators including *Lcn2, S100a8*, and *S100a9*. The anti-inflammatory effect of PF4 showed age dependence, with no detectable effect in young mice, suggesting PF4 exerts modulatory effects preferentially in aging-associated inflammatory contexts.

### 3.3 PF4 enhances synaptic plasticity

Impaired synaptic plasticity represents a well-established mechanism underlying age-related cognitive deficits (44). PF4 treatment significantly increased the expression of synaptic plasticity-related genes in the hippocampus of aged mice, including Bdnf, Ntf3, and Tmem108 (11). The cAMP response element-binding protein (CREB) represents a well-characterized transcription factor governing synaptic plasticity and memory formation. Immunohistochemical analysis showed that PF4 treatment significantly elevated the level of CREB phosphorylated at Ser133 (p-CREB) in the dentate gyrus of the aged mouse hippocampus. This finding is highly consistent with the report by Villeda et al. that young blood enhances hippocampal CREB phosphorylation in aged mice (8).

Within the central nervous system, PF4 facilitates hippocampal synaptic plasticity via GluN2B-containing N-methyl-D-aspartate receptor (NMDAR) signaling cascades. Park et al. used ex vivo electrophysiological recordings to demonstrate that direct application of PF4 to hippocampal slices significantly enhanced long-term potentiation (LTP), an effect that was completely blocked by the NMDAR receptor antagonist Ro25-6981 (12). Systemic PF4 treatment also significantly enhanced LTP in hippocampal slices from aged mice, indicating that PF4 can improve synaptic function at the organismal level.

Systemically delivered PF4 improves cognitive function via two distinct, complementary pathways, yet published reports present contradictory results regarding PF4’s capacity to traverse an intact blood-brain barrier (BBB). Park et al. detected His-tagged recombinant PF4 within hippocampal parenchyma following peripheral administration, with fluorescent signal concentrated in the dentate gyrus and spatially separated from cerebral vasculature, which suggests circulating PF4 may cross the BBB to interact with central neural tissue. Conversely, a separate study employing HiBiT-tagged PF4 detected negligible cerebral PF4 signal after systemic injection, even as PF4 treatment still rescued age-associated cognitive deficits. This latter work prioritizes a peripheral immune-centric mechanism: PF4 remodels aged immune cell populations, lowers systemic concentrations of pro-senescent inflammatory mediators, and limits inflammatory cell infiltration into the hippocampus to reduce neuroinflammation without central PF4 accumulation (11). Taken together, preclinical data support two non-mutually exclusive mechanisms underlying PF4’s neuroprotective phenotypes: putative direct central signaling upon brain entry, and indirect neuroprotection mediated by peripheral immune reprogramming.

### 3.4 PF4 promotes hippocampal neurogenesis

Adult hippocampal neurogenesis plays an important role in learning, memory, and mood regulation, and its significant decline with age is a major contributor to cognitive aging (44). Leiter et al. demonstrated that PF4 treatment markedly increased the number of doublecortin (DCX)-positive immature neurons in the aged mouse dentate gyrus, whereas Ki67-positive proliferating cell numbers showed no significant change. This indicates PF4 primarily modulates later stages of neurogenesis—specifically the survival and maturation of newborn neurons—rather than early proliferative events (13). In the PF4 treated group, the total dendrite length of DCX- positive neurons was significantly increased, and the lengths of primary dendrites and the longest dendrite were also significantly elongated. In PF4 knockout mice, the primary dendrite length of DCX-positive neurons was significantly shorter than that of wildtype controls, further confirming a contributory role of PF4 in dendritic morphogenesis.

**Figure 1:**
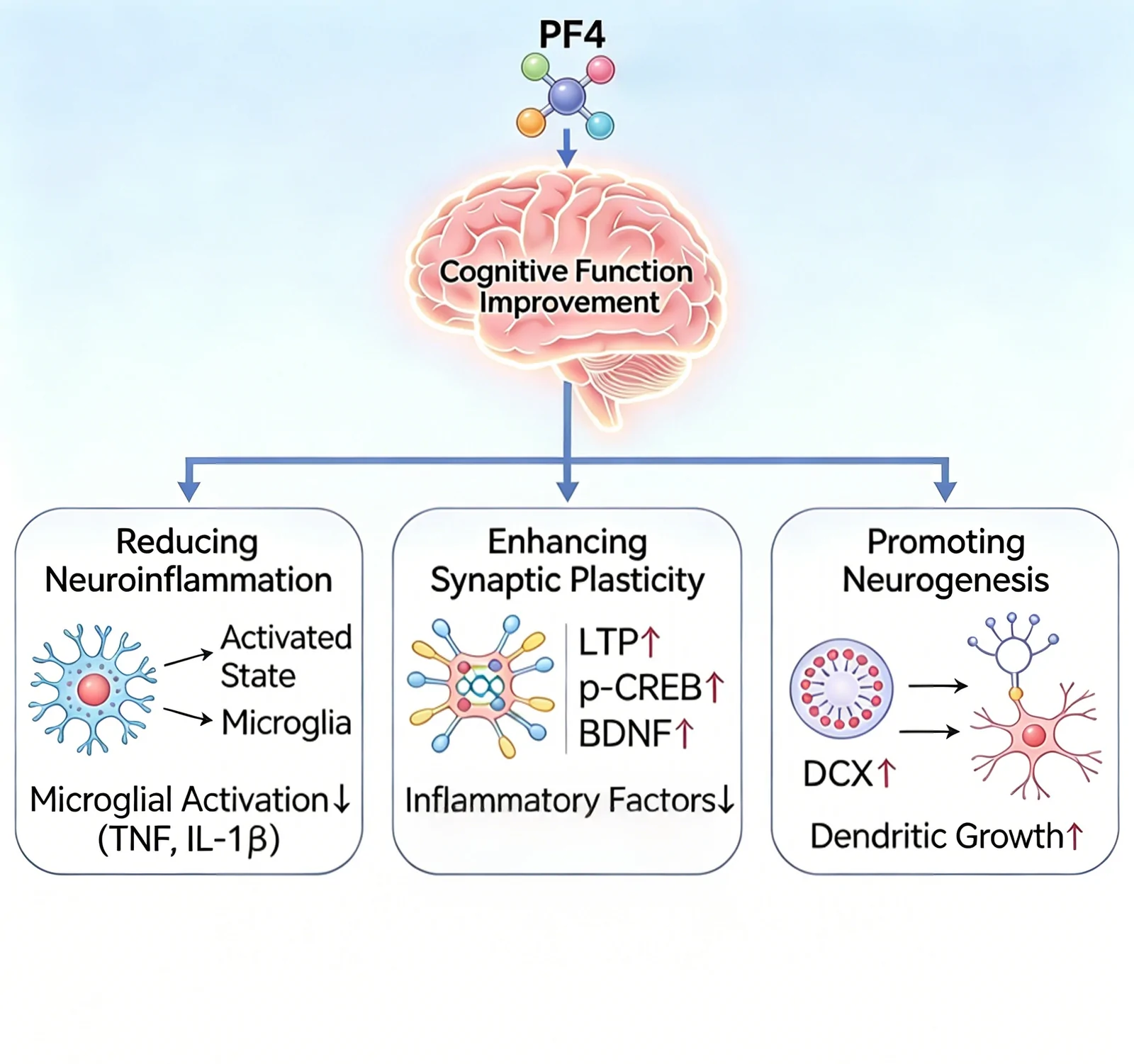
Core mechanisms of PF4 in improving cognitive aging. This schematic diagram systematically illustrates three core pathways through which platelet factor 4 (PF4) ameliorates age-related cognitive decline. PF4 exerts its cognitive-enhancing effects through multi-target synergistic actions: (1) alleviating hippocampal neuroinflammation by inhibiting microglial overactivation and downregulating the expression of pro-inflammatory cytokines such as TNF and IL-1β, thereby reversing the chronic low-grade inflammatory state of the aged brain; (2) enhancing synaptic plasticity by upregulating key molecules including long-term potentiation (LTP), phosphorylated CREB (p-CREB), and brain-derived neurotrophic factor (BDNF), thereby improving synaptic function and signal transmission; and (3) promoting hippocampal neurogenesis by increasing doublecortin (DCX) expression and facilitating the survival, maturation, and dendritic growth of newborn neurons. Ultimately, the synergistic action of these three pathways comprehensively improves cognitive function in aged individuals.

*In vitro* experiments confirmed that PF4 could be taken up by neural precursor cells, with intracellular fluorescent signal markedly enhanced within 2–6 hours. Under differentiation conditions, PF4 treatment significantly increased the proportion of β-III-tubulin-positive neurons but had no significant effect on the proportion of GFAP-positive astrocytes, indicating that PF4 promotes the differentiation of neural precursor cells toward the neuronal lineage rather than toward the glial lineage. Notably, the pro-neurogenic effect of PF4 is brain-region specific: it is prominent on neural precursor cells in the hippocampal dentate gyrus but shows no obvious effect on those in the subventricular zone. This finding is highly consistent with the differential effects of exercise on these two neurogenic niches, further supporting PF4 as a partial contributor to exercise-induced hippocampal neurogenesis (45). Accordingly, the core mechanisms by which PF4 improves cognitive aging are summarized in **Figure 1**.

## 4 PF4 and age-associated alterations of the peripheral immune system

### 4.1 Immunosenescence

Immunosenescence represents a well-characterized hallmark of aging, defined by thymic involution, diminished naive T-cell output, memory T-cell accumulation, and sustained chronic low-grade inflammation (42). Beyond adaptive T cell remodeling, aging profoundly reshapes innate immune compartments, which constitute the first line of host defense and dominate the onset of inflammaging. Age-related functional defects affect multiple innate immune subsets: circulating monocytes and tissue-resident macrophages acquire exaggerated pro-inflammatory priming and enhanced SASP secretion; neutrophils show diminished chemotaxis, phagocytosis, and pathogen clearance; natural killer (NK) cells exhibit impaired cytotoxicity against senescent cells; and dendritic cells lose antigen-presenting efficiency, collectively exacerbating inflammatory dysregulation. All these dysfunctions of innate immunity synergize with adaptive immunosenescence to fuel chronic systemic inflammation. Immunosenescence is an important driver of systemic aging; through SASP and loss of immune cell function, it directly drives aging and damage in solid organs. Transplantation of senescent immune cells induces systemic aging in young mice, whereas transplantation of young immune cells suppresses aging (46).

### 4.2 PF4 reshapes peripheral immune cell composition

Schroer et al. used single-cell transcriptome and epitope sequencing (CITE-seq) to perform a comprehensive analysis of splenic immune cells in aged mice (11). The ratio of myeloid to lymphoid cells was significantly elevated in the spleens of aged mice, and PF4 treatment restored it to young levels. Within the myeloid cell population, PF4 restored a younger gene expression signature, including downregulation of type I interferon signaling, the inflammatory mediators Lcn2, S100a8, S100a9, and the anaphylatoxin complement component C3.

Notably, PF4 exerts profound multifaceted regulatory effects on T cell subsets, which constitute the core adaptive immune compartment altered during aging. Within the T-cell population, PF4 treatment significantly reduced the expression of the cytotoxicity marker Nkg7 and the exhaustion marker Tox, and genes associated with naive T-cell phenotype were restored to more youthful expression levels. At the transcriptional level, PF4 globally suppressed aging-induced pro-exhaustion transcriptional programs in T cells, while upregulating key homeostatic and naive-state transcription factors that maintain intact T cell function. Single-cell clustering analysis showed that PF4 treatment significantly reduced the proportion of age-related terminally differentiated effector memory T cells with robust pro-inflammatory profiles, while elevating the proportions of functionally quiescent naive T cells and central memory T cells with superior self-renewal capacity. Functionally, aged effector memory T cells exhibit sustained secretion of pro-inflammatory cytokines and impaired proliferative responsiveness upon antigen stimulation; PF4 intervention reverses this aged functional defect, restores balanced T cell subset distribution, and alleviates T cell-driven systemic inflammaging. Collectively, these preclinical findings demonstrate that PF4 counteracts age-associated T-cell differentiation skewing and attenuates progressive T-cell exhaustion to restore balanced adaptive immune profiles in mice.

Mechanistically, PF4-CXCR3 signaling may suppress NF-κB nuclear translocation, thereby transcriptionally repressing the senescence-associated secretory phenotype (SASP) in both microglia and peripheral monocytes. Further investigations are warranted to clarify whether PF4 modulates NOD-like receptor family pyrin domain-containing 3 (NLRP3) inflammasome activation and the release of neutrophil extracellular traps (NETs) during inflammaging.

### 4.3 PF4 reduces circulating levels of pro-senescent mediators

Accumulation of pro-inflammatory and pro-senescent mediators in the aged circulation represents a well-documented mechanism driving multiorgan aging. Age-related declines in hippocampal neurogenesis and cognitive function can be partially attributed to alterations in circulating blood-borne factors (47). PF4 administration significantly reduces plasma levels of the pro-senescent mediators CCL2, cyclophilin A (CyPA), and TNF-α in aged mice (11). These mediators have been shown to compromise blood-brain barrier integrity, suppress hippocampal neurogenesis, and impair cognitive function (48, 49). Downregulation of these circulating mediators by PF4 is thought to contribute to its neuroprotective phenotype.

### 4.4 CXCR3 mediates the peripheral immunomodulatory effects of PF4

CXCR3 is one major functional receptor for PF4. It is highly expressed on peripheral immune cells especially T cells and myeloid cells but is expressed at very low levels in brain parenchymal cells. In aged Cxcr3-knockout mice receiving systemic PF4 treatment, the peripheral immune rejuvenation triggered by PF4 was largely abrogated. Specifically, PF4 failed to reverse the age-associated elevation in the splenic myeloid-to-lymphoid cell ratio; meanwhile, the capacity of PF4 to suppress the expression of T cell exhaustion markers such as Nkg7 and Tox was substantially blunted, demonstrating that CXCR3 is required for full peripheral immune remodeling by PF4 (11). However, some effects such as the improvement in the Y-maze test remained present in CXCR3 knockout mice, suggesting that PF4 may also act through CXCR3-independent mechanisms.

## 5 PF4 and aging-related phenotypes of hematopoietic stem cells

### 5.1 Hematopoietic stem cell aging

Aging of hematopoietic stem cells (HSCs) acts as a major driver of immunosenescence and age-associated hematological disorders. Aged HSCs display characteristic pathological features: expanded cellular pools with impaired function, diminished lymphoid differentiation potential with myeloid skewing, compromised self-renewal capacity, accumulated DNA damage, and epigenetic reprogramming (50, 51).

### 5.2 Regulation of HSCs by PF4

The bone marrow microenvironment (niche) is critical for HSCs maintenance and functional regulation. Among the various niche cell types, megakaryocytes (MKs) maintain HSC quiescence by secreting PF4 and TGF-β (52). Bruns et al. first characterized PF4’s role in preserving HSC quiescence: PF4 administration markedly suppresses HSC proliferation, while PF4-deficient mice display expanded HSC populations and impaired quiescence (39). Zhang et al. reported that aged mice exhibit increased bone marrow MK numbers but reduced MK size, along with a markedly decreased proportion of polyploid MKs. RNA-seq showed that in aged MKs, immune and inflammation-related genes were upregulated, while differentiation- and function-related genes such as Pf4 were downregulated (24).

### 5.3 PF4 alleviates aging-associated HSC abnormalities

Continuous PF4 delivery via osmotic pumps over 42 days normalized the abundance of phenotypic bone marrow HSCs in 22-month-old mice to levels comparable to young animals and elevated the fraction of HSCs arrested in the G0 quiescent phase (24). This observation aligns with PF4’s suppressive action on HSC proliferative activity. Immunofluorescence staining against γH2AX, a canonical marker of DNA lesions, revealed diminished DNA damage signals and recovered normal cell polarity in PF4-treated HSCs from aged mice. qPCR quantification demonstrated that PF4 downregulated myeloid (Fgr, Osmr, Rab27b) and megakaryocyte/platelet lineage transcripts (Clu, Itgb3) in aged HSCs while upregulating lymphoid lineage genes (Mtcp1, Blnk), shifting HSC transcriptional signatures away from aged expression patterns. Competitive transplantation assays demonstrated that HSCs isolated from PF4-administered aged mice achieved superior donor chimerism and enhanced lymphoid production 4 months after primary transplantation, and this functional benefit was retained in secondary transplant recipients. Follow-up ex vivo cultures of human CD34^+^ hematopoietic stem cells also identified multiple corrected aging-related cellular features following PF4 treatment. Notably, all the above beneficial hematopoietic phenotypes have only been validated in *in vivo* murine models and human ex vivo culture systems. To date, no *in vivo* human studies have demonstrated sustained long-term therapeutic or transplant-related benefits of PF4 administration.

### 5.4 LDLR and CXCR3 mediate PF4 regulation of HSCs

**Figure 2:**
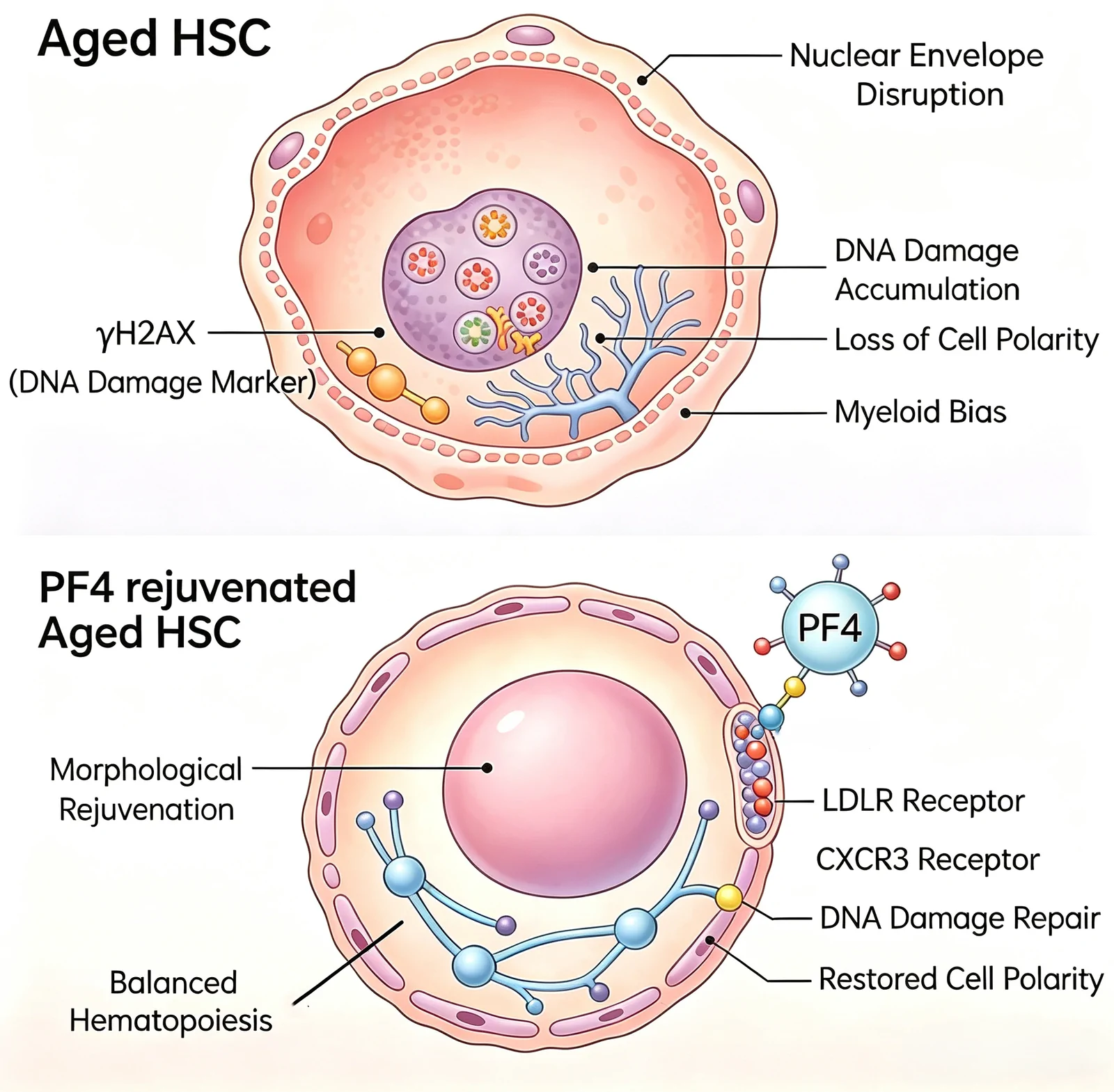
Effects of PF4 treatment on aged hematopoietic stem cells (HSCs). Schematic comparison between aged hematopoietic stem cells and PF4-treated aged HSCs. Aged HSCs (upper panel) exhibit typical aging characteristics, including elevated DNA damage markers (γH2AX), loss of cell polarity, and myeloid differentiation bias. Following PF4 treatment (lower panel), PF4 binds to its surface receptors (LDLR and CXCR3) on HSCs, reduces DNA-damage-associated γH2AX, restores cell polarity, and partially rescues youthful hematopoietic differentiation balance.

LDLR and CXCR3 represent the two primary receptors mediating PF4’s inhibitory effect on HSC proliferation (24). *Ldlr^-^/^-^; Cxcr3^-^/^-^* double-knockout mice develop premature HSC aging phenotypes by 3 months of age, including expanded HSC pools, pronounced myeloid skewing, accumulated DNA damage, and loss of cell polarity. Mechanistically, PF4 suppresses LDL uptake by HSCs in a dose-dependent manner, indicating PF4 modulates HSC fate via LDLR-dependent lipid metabolic pathways. The regulatory effects of PF4 on aging-related hematopoietic stem cell phenotypes are summarized in **Figure 2**.

## 6 Association of PF4 with Klotho, exercise, and young blood

### 6.1 The Klotho–PF4 axis

Klotho is a longevity-extending factor, a transmembrane protein mainly expressed in the kidneys; its overexpression extends mouse lifespan by approximately 30%, whereas Klotho-mutant mice exhibit a progeroid syndrome (53). The extracellular domain of Klotho can be proteolytically cleaved and released into the circulation, exerting hormone-like systemic effects. However, owing to its large molecular size (~130 kDa), full-length Klotho cannot efficiently traverse the intact blood–brain barrier; the mechanism by which it modulates central nervous system function has therefore long remained incompletely understood (54).

Soluble circulating Klotho exerts systemic aging-modulatory effects via multiple peripheral effector cell populations, and platelets have been identified as one key cellular intermediate linking Klotho signaling to brain function. *In vitro* and *in vivo* data from Park et al. demonstrated that soluble Klotho induces mild, non-thrombotic platelet activation without changing total platelet counts. This moderate activation does not trigger pathological thrombosis but selectively promotes α-granule degranulation, releasing abundant PF4 and other platelet-derived bioactive factors (12). Mechanistically, soluble Klotho weakly interacts with platelet surface receptors to amplify ADP-dependent platelet signaling cascades, driving moderate degranulation; this regulatory process is independent of canonical FGF23/FGFR mineral metabolism signaling, indicating a novel aging-modulatory function separate from Klotho’s classic renal endocrine roles. Functionally, Klotho-induced platelet degranulation and PF4 release broadly counteract inflammaging: as detailed in earlier sections, PF4 suppresses overactivation of peripheral myeloid cells, attenuates chronic low-grade systemic inflammation, and rectifies age-related immune cell subset skewing, thereby indirectly alleviating neuroinflammation and restoring hippocampal synaptic plasticity to ameliorate age-dependent cognitive decline. *In vivo* intervention studies by Park and colleagues further support the existence of a Klotho–platelet–PF4 signaling axis, with key evidence summarized below.

Klotho treatment significantly increased plasma PF4 levels in mice and increased the percentage of CD62P-positive platelets, a marker of platelet activation. Pretreatment with aspirin and clopidogrel completely blocked the cognitive-enhancing effect of Klotho and also blocked the Klotho-induced rise in plasma PF4. Peripherally administered His-tagged PF4 was detected within the brain parenchyma, with prominent signal observed in the hippocampal dentate gyrus, consistent with possible brain entry across the blood–brain barrier; electrophysiological experiments confirmed that direct application of PF4 to hippocampal slices enhanced LTP. At the behavioral level, PF4 partially recapitulates the cognitive benefits of Klotho; notably, Klotho remains functionally active in PF4-knockout mice, confirming that PF4 may contribute to, but is not required for, the cognitive effects induced by Klotho.

### 6.2 The exercise–PF4 axis

Physical activity is a well-documented non-pharmacological intervention that enhances hippocampal neurogenesis and cognitive function (55). Initial work by Leiter et al. demonstrated that exercise-induced platelet activation boosts adult hippocampal precursor proliferation and neuronal differentiation; their follow-up studies further identified platelets and PF4 as key mediators of this effect (13, 56).

As little as 4 days of voluntary wheel running significantly increased plasma PF4 levels in mice, and the percentage of CD62P-positive activated platelets increased significantly after exercise, returning to baseline after 7 days of exercise, indicating that exercise-induced platelet activation is an acute, transient response (13). Exercise significantly increased the number of Ki67-positive proliferating cells in the dentate gyrus of control mice, whereas platelet-depleted mice showed no significant change in Ki67-positive cell numbers after exercise. In PF4 knockout mice, exercise-associated neurogenesis was fully abrogated. These findings support a contributory role for PF4 in exercise-stimulated hippocampal neurogenesis. *In vitro* assays confirmed that PF4 increases neurosphere formation from hippocampal neural precursor cells and promotes neuronal differentiation. Consistent with observations detailed in Section 3.4, local hippocampal PF4 infusion increases DCX-positive immature neuron numbers, indicating PF4 primarily modulates later stages of neurogenesis (survival and maturation).

### 6.3 The young blood–PF4 axis

The cognitive-enhancing effect of young blood on the aged brain was first reported by Villeda et al., who showed that repeated intravenous injection of young mouse plasma into aged mice reversed molecular, structural, functional, and cognitive aging deficits in the aged brain and improved hippocampal-dependent learning and memory (8). However, which factor in young blood mediates this effect long remained unresolved.

The study by Schroer et al. provided a key answer (11). Conventionally prepared young plasma actually contains large numbers of platelets; when platelets were removed by centrifugation, the cognitive-enhancing effect of young plasma completely disappeared, whereas the platelet-rich plasma fraction retained full cognitive-improving ability, indicating that platelets are an essential cellular component for the cognitive-enhancing effect of young blood. Further analysis showed that PF4 levels in plasma from young mice and young humans are significantly higher than those in aged individuals. Administering exogenous PF4 alone to aged mice alleviated hippocampal neuroinflammation, promoted synaptic plasticity, and improved cognitive function.

**Figure 3:**
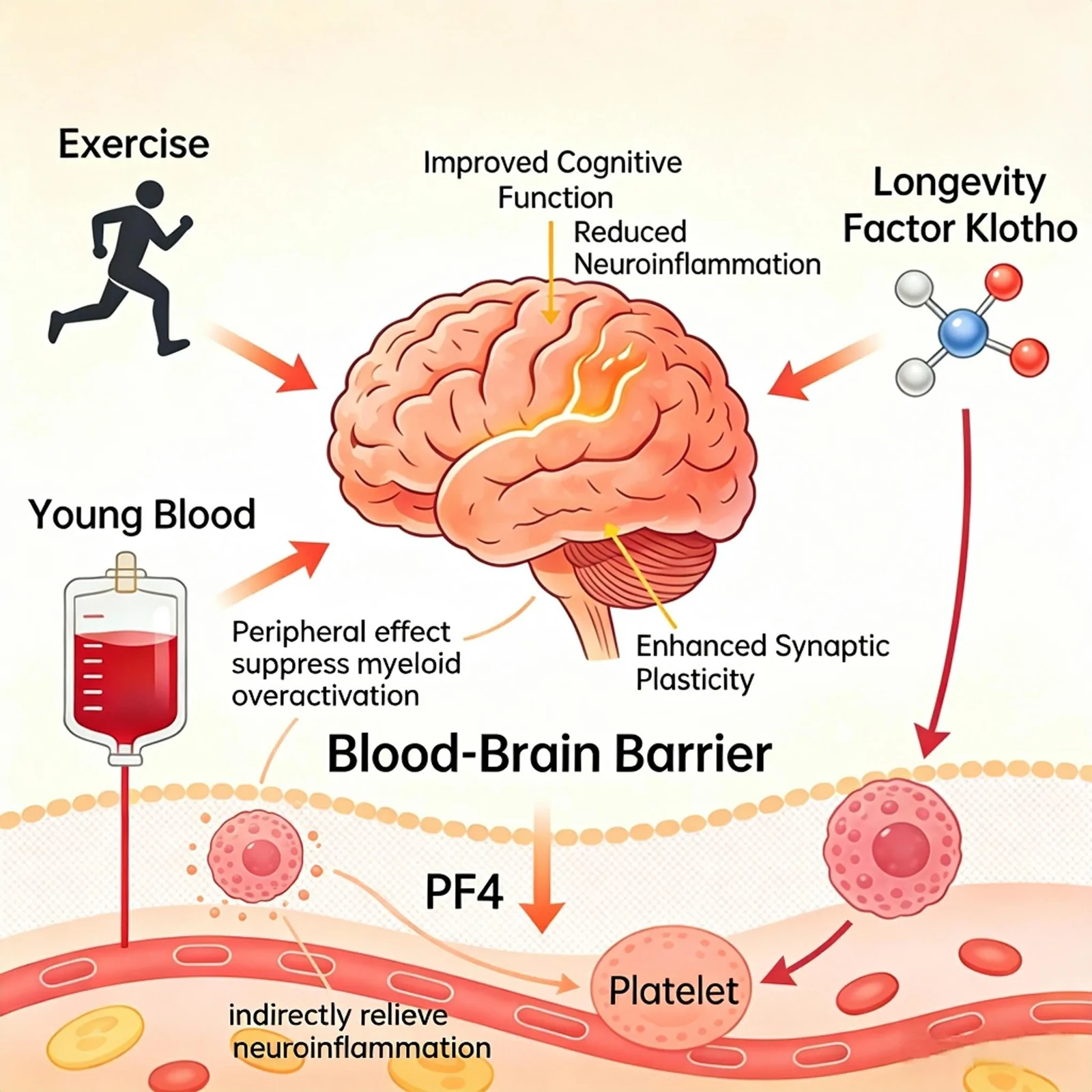
Convergence model of three anti-aging interventions targeting platelet-derived PF4 to restore hippocampal and cognitive function. This schematic illustrates that Klotho, exercise and young blood all trigger platelet activation and the release of circulating PF4. PF4 may exert dual anti-aging effects: peripherally, it suppresses systemic inflammaging to indirectly alleviate brain neuroinflammation; centrally, PF4 could gain access to the brain to modulate hippocampal function, enhancing synaptic plasticity, facilitating neurogenesis and improving learning and memory. Platelet-derived PF4 represents a potential partial mediator contributing to the neuroprotective benefits associated with these three interventions that alleviate age-related functional decline.

Collectively, these findings suggest PF4 may act as a partial shared mediator downstream of several rejuvenation interventions, consistent with a three-path convergence framework depicted in **Figure 3**. PF4 may function as one shared partial intermediate downstream of three interventions that mitigate aging-related functional decline, providing a candidate molecular scaffold for developing therapeutics against age-associated cognitive deficits.

### 6.4 The unique value of PF4 as a partial exercise mimetic

Exercise, Klotho, young blood and PF4 can modulate cognitive aging linked to the PF4 axis, yet the four interventions differ substantially in efficacy, acting spectrum, safety and clinical accessibility. Simply emphasizing that PF4 is a common downstream molecule risks oversimplification-exercise has systemic benefits that PF4 cannot replace, while young blood and Klotho face different translational obstacles. A systematic comparison of these four strategies across multiple dimensions is necessary to more rigorously define the translational value of PF4.

Physical activity has well-documented systemic benefits, favorable safety profiles, and broad physiological effects across multiple organ systems. While exercise-induced hippocampal neurogenesis appears to be largely PF4-dependent, other effects such as cardiovascular adaptation and improved peripheral metabolism are entirely independent of PF4 (13). Accordingly, PF4 should be defined strictly as a partial exercise mimetic restricted to cognitive aging and hippocampal neurogenesis and cannot recapitulate the full spectrum of systemic health benefits conferred by physical activity.

PF4 has unique advantages over Klotho and young blood. Although Klotho activates platelets to release PF4, its cognitive-enhancing effect persists in PF4-knockout mice, suggesting that Klotho also acts through other redundant pathways like direct effects on peripheral nerves, making its mechanism more complex (12). Young blood contains multiple factors, and its synergistic effects are difficult to deconstruct. In contrast, PF4 is a single molecule, making it easier to achieve standardized production and pharmacokinetic control. The manufacturing process for recombinant PF4 is well established, such as clinical-grade PF4 used for HIT diagnosis, and its safety can be optimized through engineered variants, whereas young blood faces ethical and regulatory hurdles, and Klotho has a very short half-life (approximately 10 minutes), limiting its application.

Although exercise-related improvements in hippocampal neurogenesis appear largely reliant on PF4, PF4 may represent a promising candidate cognitive protectant or neuroprotective agent for elderly populations unable to engage in exercise owing to physical constraints, such as individuals bedridden following fracture and patients with advanced Alzheimer’s disease or severe sarcopenia. It should not be viewed as a substitute for exercise. Future work could explore combinations of PF4 with low-intensity exercise or other exercise mimetics, which may cooperatively alleviate diverse aging-related phenotypes.

## 7 Clinical translational potential of PF4

### 7.1 Clinical relevance

Notably, all clinical data linking PF4 to aging-associated disorders originate from cross-sectional observational studies. These findings only demonstrate statistical associations between circulating PF4 levels and disease status and cannot establish causal relationships or confirm therapeutic efficacy. Nevertheless, emerging clinical evidence has gradually elucidated the relevance of PF4 to human aging and neurodegenerative conditions, providing preliminary clues for future PF4-based research directions.

The clinical translational potential of PF4 is first reflected in its value as a diagnostic biomarker for various aging-associated disorders. In patients with Alzheimer’s disease, serum PF4 levels are significantly decreased and correlate significantly with cognitive function as well as with cerebrospinal fluid levels of Aβ42 and t-tau. This suggests PF4 may be involved in AD pathophysiology via anti-inflammatory and neuroprotective pathways and may represent a candidate adjunct diagnostic biomarker for AD (57). In patients with sarcopenia, the concentration of PF4 in plasma small extracellular vesicles is significantly reduced and closely correlates with muscle strength and physical performance, demonstrating good diagnostic efficacy, suggesting that PF4 may serve as a potential plasma biomarker and may be involved in the pathogenesis of this disease (58). Furthermore, in patients with hemorrhagic stroke, blood PF4 levels are significantly elevated and have been validated as a potential biomarker, suggesting potential diagnostic utility for rapid stroke identification (59). In patients with acute coronary syndrome, PF4 levels are elevated in those who experience major adverse cardiovascular events; its high sensitivity (98%) makes it a potential screening marker for identifying early thrombotic risk, but it is not an independent predictor and should be used in combination with other inflammatory, coagulation, and autoimmunity markers to improve prognostic assessment (60).Recent population-based evidence also indicates reduced circulating PF4 concentrations in patients with amyotrophic lateral sclerosis (ALS), further linking circulating PF4 to human neurodegenerative disorders (61).

Notably, all above evidence originates from cross-sectional clinical surveys, which only demonstrate statistical correlations between PF4 concentrations and disease status. Such observational data are insufficient to verify causal links, reliable diagnostic utility, or definite therapeutic effects of PF4 in human age-related disorders.

### 7.2 Neuroprotective effects

Studies have found that PF4 has dual effects: protecting the blood–brain barrier in ischemic stroke and inhibiting ferroptosis in intracerebral hemorrhage. In ischemic stroke, using an intermittent hypoxic preconditioning model, PF4 acts as a key paracrine mediator induced by intermittent hypoxia. Exogenous administration of PF4 or plasma from intermittently hypoxic mice protects blood-brain barrier integrity, by reducing post-ischemic IgG leakage, maintaining endothelial tight junction protein ZO-1 expression. It also reduces cerebral infarct volume and improves neurological function; these protective effects are abolished in the absence of PF4 (62). In an intracerebral hemorrhage model, PF4 expression is significantly downregulated. Supplementation with PF4 inhibits neuronal ferroptosis through the CXCR3/PI3K/AKT/Nrf2 signaling pathway, thereby reducing brain edema, improving neurological function, and attenuating neuronal damage (22). Beyond acute brain injury models, emerging preclinical work extends PF4’s neuroprotective function to chronic degenerative motor neuron disease. Systemic administration of recombinant PF4 in hSOD1^G93A^ mice produced dramatic therapeutic effects: extended survival, preserved motor function, attenuated neuroinflammation, and reduced neuromuscular junction denervation. Mechanistically, exogenous PF4 triggers LRP1-TBK1-OPTN-dependent autophagy to clear toxic superoxide dismutase 1 (SOD1) aggregates (61).

Collectively, these preclinical findings support a plausible hypothesis that PF4 serves as an endogenous neuroprotective mediator with broad protective effects in both acute brain injury and chronic neurodegenerative disorders. Although current animal data are encouraging, the translational potential of PF4 remains speculative and requires extensive further validation before it can be established as a viable therapeutic target or clinical candidate for neurological diseases.

### 7.3 Potential for exogenous intervention

In the context of hematopoietic stem cell research, Zhang et al. reported that recombinant human PF4 could promote quiescence in bone marrow CD34^+^ cells derived from young, middle-aged and aged human donors, regardless of age, while alleviating accumulated age-associated DNA lesions and recovering normal cell polarity (24). This indicates that the aging-modulatory effects of PF4 on HSCs are not restricted to specific age groups and supports further exploration of PF4 for ex vivo manipulation of aged HSCs. Potential applications include preconditioning of HSCs from older donors to improve engraftment outcomes in preclinical transplant models, or as a preclinical intervention strategy for age-related hematopoietic dysfunction.

In an animal model of cognitive impairment induced by thyroxine, the disease state was characterized by significant systemic and neuroinflammation, and PF4 levels were relatively low. Treatment with proanthocyanidin supplementation significantly elevated PF4 levels, restoring them to near-normal levels. The increased PF4 acted as an anti-inflammatory mediator, directly or indirectly inhibiting the production and release of multiple pro-inflammatory factors in the blood. This effect effectively ameliorated the systemic inflammation induced by thyroxine (63). These findings provide a preliminary theoretical basis for the speculative hypothesis that modulating PF4 through dietary supplements such as proanthocyanidins may serve as a potential strategy for intervening in age-related cognitive impairment, though future validations are still essential.

### 7.4 Age-related controversy and implications

Regarding age-related alterations in PF4 concentrations, published clinical observations remain inconsistent across studies. One cohort reported significantly higher PF4 concentrations in platelet-rich plasma (PRP) from young participants (mean age 27.1 years) relative to elderly subjects (mean age 66.3 years) (11). Consistently, Weng et al. demonstrated elevated PF4 levels in PRP lysates isolated from young peripheral blood, and qPCR analysis across young adults, aged adults and umbilical cord blood confirmed maximal PF4 transcript abundance in young peripheral blood cells (64).

In contrast, Duchez et al. reported opposite results based on analysis of apheresis platelet concentrates, showing that platelet products collected from aged donors contained higher PF4 content than those from younger donors, with PF4 concentrations gradually declining during storage (65). As summarized herein, multiple preanalytical and technical variables may substantially account for these inter-study discrepancies, including differences in sample matrices (serum, PRP, platelet-poor plasma, platelet lysates, isolated platelet vesicles), baseline platelet counts, ex vivo platelet activation status, centrifugation parameters, storage duration, and standardized sample processing protocols. Variations in participant age stratification and confounding background medication history may further hinder direct cross-study comparison of human PF4 measurements.

Of note, we emphasize that the dominant cause of these inconsistent findings (technical artifacts versus inherent biological, age-dependent PF4 regulation) remains not fully established. It is hypothesized rather than conclusively determined that such technical discrepancies do not negate a potential physiological association between PF4 and organismal aging. Notably, age-related PF4 alterations may exist in a context-, sample-, or disease-dependent manner, which could partially explain the conflicting human observations.

Furthermore, studies have failed to reach a unified conclusion on the change of intracellular PF4 storage in aged platelets. Aging may affect platelet degranulation and subsequent PF4 signal transduction, which renders exogenous PF4 supplementation a research direction worthy of follow-up validation.

## 8 Challenges and perspectives of PF4

PF4 exhibits encouraging effects against several age-related pathological phenotypes, yet relevant research remains in the preliminary stage, with numerous obstacles hindering clinical translation. First, major safety hurdles stem from PF4’s multifaceted pleiotropic bioactivity *in vivo*. PF4 governs hemostatic signaling; while it confers tissue-protective impacts on aged tissues in animal models, it concurrently carries thrombogenic risks. The complex formed by PF4 and heparin is the major antigen in HIT, and various negatively charged molecules *in vivo* may form antigenic complexes with PF4. High concentrations of PF4 can directly activate platelets. PF4 binds to the c-Mpl receptor on the platelet surface, activating the JAK2-STAT3/5 pathway and inducing platelet aggregation (29). In the elderly population, where baseline platelet activation is already elevated, the additive effect of PF4 may further increase the risk of thrombotic events. Strategies to mitigate and monitor PF4-associated thrombotic hazards represent a critical unresolved barrier before any human translational testing can proceed. Hypothetical mitigation strategies include engineering PF4 variants that preserve CXCR3 binding while lowering heparin affinity. Future translational work must prioritize the optimization of dosing regimens and thrombotic-risk surveillance to balance therapeutic benefits and safety. All such schemes remain purely theoretical and demand exhaustive preclinical validation prior to human testing.

All existing frameworks framing PF4 as a therapeutic candidate for aging-related dysfunction, alongside proposed patient cohorts and antiplatelet combinatorial regimens, remain highly preliminary and lack human supporting data. Extensive preclinical profiling is urgently needed, encompassing pharmacokinetic profiling, dose-response titration, long-term chronic toxicology, and systematic risk evaluation of thrombosis, organ fibrosis, anti-PF4 autoantibody generation, and tissue-specific safety readouts.

A secondary safety liability of PF4 is its profibrotic activity, which has been validated across preclinical models of systemic sclerosis, hepatic, bone marrow, and cardiac fibrosis (66–68). The mechanisms involve activation of the TGF-β signaling pathway, promotion of fibroblast to myofibroblast transition, and recruitment of activated macrophages. To address the potential fibrotic risk, intermittent dosing, combination with anti-fibrotic drugs, and optimized therapeutic windows are tentative strategies requiring further preclinical validation.

An additional safety issue is the role of PF4 as an autoantigen. In HIT and VITT, anti-PF4 antibodies activate platelets, monocytes, and neutrophils via FcγRIIA, leading to thrombocytopenia and thrombosis (69). Although most anti-PF4 antibodies do not possess platelet-activating activity, unpredictable antibody generation remains a potential risk. Coping strategies include using human PF4, screening for anti-PF4 antibodies before treatment, and developing PF4 mimetic peptides that avoid major B-cell epitopes. Other avenues to explore are the use of platelets as natural carriers for targeted PF4 release, as well as PF4 overexpressing mesenchymal stem cells or engineered extracellular vesicles. It may also be valuable to develop a predictive model for cognitive decline risk based on PF4 levels, and to monitor anti-PF4 antibody production during treatment.

Second, all available PF4 intervention studies rely on short-term dosing paradigms in animal models; long-term biological consequences remain entirely uncharacterized in any human cohort. Recombinant PF4 has been used in early-phase clinical trials to reverse the anticoagulant effect of heparin, with no serious adverse events observed, but the sample sizes were small and treatment durations short, insufficient to assess long-term safety. Given the limited short-term animal-only dataset, direct clinical deployment of PF4 to treat aging-associated dysfunction cannot be justified at this stage. Research priorities should include long-term toxicology assays in non-human primates, high-throughput screening of modified PF4 variants, and preclinical testing of combinatorial treatment regimens. The long-term effects of PF4 on cognition, the immune system, the hematopoietic system, and even lifespan still require further investigation.

Nevertheless, several key questions remain, including the potential role of PF4 in blood brain communication, its interactions with neurotransmitters and neuropharmacological processes, and how to translate these findings into clinical practice. More detailed studies are needed to validate and extend these insights for therapeutic applications (70). It has been shown that optimized composite microsphere hydrogels can serve as an amplifier for intranasal brain delivery of PF4, targeting human senescent cells induced by anesthesia and surgery to repair neurocognitive dysfunction (41). Although new approaches such as intranasal administration offer promise, the optimal route of administration, dosage, frequency, and therapeutic window still need to be optimized in clinical studies. Currently, standardized pharmacokinetic data for PF4 are sparse across different animal models. However, the available evidence suggests that heparin-binding affinity drastically alters PF4 bioavailability, and its rapid binding to extracellular matrix proteoglycans may limit systemic exposure. Moreover, the short *in vivo* half-life of recombinant proteins and unknown oral bioavailability constitute major translational hurdles. Future strategies including polyethylene glycol modification (PEGylation), nanoparticle encapsulation and intranasal delivery warrant further exploration to prolong PF4 half-life and improve brain targeting efficiency.

Critical caveat: nearly all mechanistic and functional evidence supporting PF4’s aging-modulatory effects derives from short-term murine assays with narrow functional readouts spanning cognition, neuroinflammation, neurogenesis, and hematopoiesis. A clear distinction must be made between PF4’s capacity to ameliorate isolated aging-linked cellular defects versus broad systemic restoration of organismal youthful function or extended health/lifespan. To date, no robust data confirm PF4 confers widespread organism-wide protection against aging; extended animal longitudinal studies and prospective human clinical cohorts will be necessary to fully delineate its aging-modulatory potential.

PF4 has shown potential therapeutic effects in neurodegenerative diseases such as AD, PD, and intracerebral hemorrhage. These initial findings call for more studies to evaluate the translational potential of PF4 in neuroscience and anti-aging. At the same time, its role in other aging-associated disorders such as atherosclerosis, osteoarthritis, and sarcopenia remains to be explored. Research exploring PF4-mediated protection against age-related pathologies spans multiple disciplines, including hematology, neuroscience, immunology, and geriatric medicine. Interdisciplinary collaboration across basic science, preclinical pharmacology, and clinical epidemiology is warranted to advance preclinical understanding of PF4’s biological roles and rigorously evaluate its translational potential.

## 9 Conclusion

PF4, a chemokine primarily secreted by platelets, is undergoing a paradigm shift from a classical coagulation regulator to a candidate molecule modulating discrete aging-related phenotypes in preclinical studies. Findings from three independent research lines focused on exercise, Klotho, and young blood collectively indicate PF4 may participate in regulating age-associated cognitive decline via peripheral and central mechanisms.

Preclinical data demonstrate PF4 exerts multi-layered regulatory effects on aging-related cellular and tissue phenotypes. Peripherally, PF4 remodels aged immune cell compartments and lowers circulating pro-senescent inflammatory factors. In the hematopoietic system, PF4 maintains HSC quiescence through LDLR and CXCR3 signaling, alleviates accumulated DNA damage, and partially restores the engraftment capacity of aged HSCs in transplant models. In the central nervous system, PF4 mitigates hippocampal neuroinflammation, improves synaptic plasticity, boosts adult hippocampal neurogenesis, and rescues age-associated cognitive deficits in mice.

However, the double-edged nature of PF4 including pro-coagulant, pro-immune-thrombotic, and pro-fibrotic demands a careful risk–benefit assessment for its clinical translation. Upstream research priorities include dissecting the refined PF4-CXCR3 signaling cascade, engineering optimized PF4 variants and mimetic peptides, designing tissue-targeted delivery platforms, and evaluating combinatorial regimens alongside other aging-modulatory mediators.

With targeted optimization of protein conformation, tissue-targeted delivery systems, and biosafety profiles, native or engineered PF4 derivatives may serve as preclinical research candidates. These molecules partially recapitulate exercise-associated neuroprotective effects and modulate age-related immune and hematopoietic dysfunction, laying a theoretical foundation for further exploration of multimodal interventions for age-associated organ impairment.
